# *In Vivo* Tracking of Systemically Administered Allogeneic Bone Marrow Mesenchymal Stem Cells in Normal Rats through Bioluminescence Imaging

**DOI:** 10.1155/2016/3970942

**Published:** 2016-08-17

**Authors:** Juan Cao, Shike Hou, Hui Ding, Ziquan Liu, Meijuan Song, Xiaojing Qin, Xue Wang, Mengyang Yu, Zhiguang Sun, Jinyang Liu, Shuli Sun, Peixin Xiao, Qi Lv, Haojun Fan

**Affiliations:** ^1^Institute of Disaster Medicine and Public Health, Affiliated Hospital of Logistic University of Chinese People's Armed Police Force, Tianjin, China; ^2^Key Laboratory of Emergency and Disaster Medicine in Chinese People's Liberation Army (PLA), Tianjin 300162, China; ^3^Department of Pathology, Affiliated Hospital of Logistic University of Chinese People's Armed Police Force, Tianjin, China

## Abstract

Recently, mesenchymal stem cells (MSCs) are increasingly used as a panacea for multiple types of disease short of effective treatment. Dozens of clinical trials published demonstrated strikingly positive therapeutic effects of MSCs. However, as a specific agent, little research has focused on the dynamic distribution of MSCs after* in vivo* administration. In this study, we track systemically transplanted allogeneic bone marrow mesenchymal stem cells (BMSCs) in normal rats through bioluminescence imaging (BLI) in real time.* Ex vivo* organ imaging, immunohistochemistry (IHC), and RT-PCR were conducted to verify the histological distribution of BMSCs. Our results showed that BMSCs home to the dorsal skin apart from the lungs and kidneys after tail vein injection and could not be detected 14 days later. Allogeneic BMSCs mainly appeared not at the parenchymatous organs but at the subepidermal connective tissue and adipose tissue in healthy rats. There were no significant MSCs-related adverse effects except for transient decrease in neutrophils. These findings will provide experimental evidences for a better understanding of the biocharacteristics of BMSCs.

## 1. Introduction

Mesenchymal stem cells (MSCs) are self-renewing, multipotent progenitor cells with the potential to differentiate into multiple mesoderm lineages. Many animal models have demonstrated the remarkable tropic, nonimmunogenic, and immunosuppressive characteristics of these cells in injured tissues [1, 2]. Due to their properties of accessibility and convenient expansion, clinical research on MSCs has increased in the past twenty years. MSCs have been described as a promising source for the cell-based treatment of miscellaneous complicated disorders, such as graft-versus-host disease [3, 4], cardiocerebrovascular disease [5], spinal cord injury [6, 7], hepatic diseases [8], and respiratory disease [9, 10]. Although there is a consensus regarding the* in vitro* characteristics of MSCs, there are outstanding issues concerning the localization and persistence of MSCs* in vivo* after administration. Furthermore, the safety of injecting of foreign cells such as these is another major obstacle in the clinical setting.

Bioluminescence imaging (BLI) detects visible light emitted by cells labeled with luminescent enzymes, such as luciferase, when these enzymes react with their specific bioluminescent substrates [11]. BLI can noninvasively track luciferase-transduced cells implanted in living animals in real time. This method of substantial utility has already facilitated the design of therapeutic strategies using MSCs in various animal models, including models of tumorous diseases, ischemia- and reperfusion-induced acute kidney injury (AKI), myocardial damage, stroke, and other diseases [12–15]. However, visible light is limited in the depth of tissue penetration. Thus, this technique is mainly used for imaging of small animals, such as mice. Herein, we show that bioluminescent imaging can reveal dynamic information detailing the distribution and tropism of BMSCs in a larger rat model. More interestingly, we revealed that the homing organs of BMSCs in rats are not the same as in mice. Additionally, we carried out a safety assessment of the administered BMSCs through histological and serology tests, providing an experimental basis for the use of these stem cells in research.

## 2. Material and Methods

### 2.1. Lentivirus Vector and BMSC Transduction

MSCs were isolated from the bone marrow of normal Wistar rats purchased from Cyagen Biosciences. According to the minimal criteria proposed by the International Society for Cellular Therapy (ISCT) [16], the cells were subjected to flow cytometry to examine the expression of specific surface antigens (Figure  S1 in Supplementary Material available online at http://dx.doi.org/10.1155/2016/3970942). The BMSCs displayed a typical MSC morphology, exhibiting a fibroblast-like shape or flat polygonal appearance. They were plastic adherent and were maintained in tissue culture flasks. When the BMSCs were approximately 80 to 90% confluent, they were dissociated with trypsin-EDTA and split at a 1 : 2 ratio.

The culture-expanded BMSCs were seeded in six-well plates at a density of 1 × 10^5^ cells per well. 24 h later, the medium was removed and replaced with growth medium and a polybrene solution (1 mL per well). Polybrene was added at a final concentration of 5 *μ*g/mL. The lentivirus vector expressing both the GFP and luciferase genes (purchased from GENECHEM, Shanghai, China) was thawed and added at a multiplicity of infection of 4. After 8 h of incubation, the medium was replaced with fresh medium, and the flask was returned to the incubator. Three days later, to quantify the transduction efficiency, Luc-GFP-BMSCs were stained with Hoechst 33342 (FANBO, Beijing, China) for 5 min. Then, the cells were visualized and analyzed using an inverted fluorescence microscope (DMI 3000B, Leica, Germany) in five randomly selected fields of view.

### 2.2. Multilineage Differentiation of Luc-GFP-BMSCs

Cultured-expanded Luc-GFP-BMSCs at passage six were used to evaluate the* in vitro* differentiation abilities of the cells in accordance with the manufacturer's recommendations (Cyagen Biosciences, USA). For adipogenic induction, Luc-GFP-BMSCs were subculture-expanded in six-well plates at 2 × 10^4^ cells/cm^2^ in growth medium containing 10% fetal bovine serum and 5% penicillin-streptomycin as well as glutamine. The cells were fed every three days until they reached 100% confluency. Then, the growth medium was changed to 2 mL of induction medium, which consisted of fetal bovine serum, penicillin-streptomycin, glutamine, insulin, rosiglitazone, and dexamethasone. Three days later, the medium was replaced with maintenance medium containing fetal bovine serum, penicillin-streptomycin, and insulin. 24 h later, the medium was changed back to induction medium, and the cycle of induction/maintenance was repeated three times. After five cycles of induction/maintenance, the cells were cultured in maintenance medium for an additional three days. Three weeks later, adipose cells were observed after being stained with Oil Red O. To induce osteogenic differentiation, Luc-GFP-BMSCs were seeded in growth medium at a density of 3 × 10^4^ cells/cm^2^ for one day at 37°C in a 5% CO_2_ humidified incubator. Then, the growth medium was aspirated from each well and 2 mL of osteogenic differentiation medium containing fetal bovine serum, penicillin-streptomycin, glutamine, ascorbate, *β*-glycerophosphate, and dexamethasone was added, and the medium was changed every three days. Three weeks later, the cells were fixed with 2 mL of a 4% formaldehyde solution and stained with Alizarin red.

### 2.3. Animals

Adult male Wistar rats weighing 170 ± 10 g were provided by the Experimental Animal Center of the Military Medical Science Academy of the People's Liberation Army of China. The rats were maintained in an animal laboratory under a temperature of 25°C at all times, fed with commercial rodent chow and given free access to water, and were allowed to acclimate for one week. All animals received humane care in compliance with the* Guide for the Care and Use of Laboratory Animals* published by the National Institutes of Health. The study protocol was approved by the Laboratory Animal Ethics Committee of the Affiliated Hospital of the Logistical College of the Chinese People's Armed Police Forces.

### 2.4. *In Vitro* Imaging

To assess the luciferase expression of the transduced BMSCs, different numbers of Luc-GFP-BMSCs (0.1, 0.2, 0.3, 0.4, and 0.5 × 10^4^/well) were seeded into a 96-well plate in 100 *μ*L of growth medium, and D-luciferin solution (D-luciferin, 150 *μ*g/mL, Gold Biotechnology, Inc., USA) was added at room temperature. After 10 min of incubation, the cells were imaged using an* in vivo* imaging system (IVIS) (PerkinElmer, IVISSPE, USA). The bioluminescent signals were analyzed using Living Image Software 4.5.

### 2.5. *In Vivo* Imaging of Luc-GFP-BMSCs

Luc-GFP-BMSCs (2 × 10^6^) suspended in 1 mL of phosphate-buffered saline (PBS) were injected into the tail vein of rats, and this group was labeled the Luc-GFP-BMSC transplantation group (Luc-GFP-BMSCs group, *n* = 5). Phosphate-buffered saline alone was used as a control. For the* in vivo* imaging of aim cells, rats were anesthetized with 2% pentobarbital sodium (50 mg/kg) and injected intraperitoneally with D-luciferin (150 mg/kg body weight) 10 min before imaging. Then, the animals were placed in the imaging chamber.* In vivo* BLI was performed at 1.5, 2.5, 18, and 22 h and 1, 2, 3, 7, 10, 14, and 30 days after Luc-GFP-BMSC injection. Regions of interest (ROIs) were drawn manually using Living Image 4.5 Software (Caliper Life Sciences) to evaluate the relative signal intensity emitted. The photon radiance of the experimental animals was expressed as photons per second per centimeter squared per steradian within the ROIs. The animals were imaged over a period of one month, after which they were sacrificed, and their tissues were harvested for PCR and IHC analyses.

To locate cell homing visually, four days after injection, immediately after acquiring photographic images* in vivo*, five animals were sacrificed. The skin of the back, the vertebral column, and other organs were removed and placed in Petri dishes. BLI of the tissues was carried to identify the Luc-GFP-BMSCs. Then, tissues showing BLI signals were fixed in 4% paraformaldehyde for further IHC analysis.

### 2.6. Immunohistochemistry

To verify the histological distribution of Luc-GFP-BMSCs after transplantation, tissues fixed in 4% paraformaldehyde were dehydrated and embedded in paraffin. Paraffin-embedded sections of 5 *μ*m were prepared on poly-L-lysine-coated slides according to standard protocols. The slides were incubated with an anti-GFP antibody (Abcam, London, England) diluted 1 : 50 in PBS overnight at 4°C. For primary antibody detection, a mouse immunohistochemistry (ABC) kit (ZSGB-BIO, Beijing, China) was used. The sections were stained with a DAB kit. Counterstaining was performed with hematoxylin. GFP-positive cells were then counted under a light microscope in six high-power fields in three sections and scored based on whether they were tissue associated.

Furthermore, we detected GFP-positive cells in tissues via IHC to verify the advanced histological distribution of Luc-GFP-BMSCs at one day after transplantation.

### 2.7. RT-PCR

Total RNA for PCR was extracted with an RNeasy kit (Solarbio Science & Technology, Beijing, China), including a DNase digestion step to exclude contaminating DNA. Reverse transcription was performed using a Quant Script kit (TIANGEN BIOTECH, Beijing, China) for 1 h at 37°C. The primer sequences for the target gene were as follows: firefly luciferase-F: ACTGGGACGAAGACGAACAC and firefly luciferase-R: GGCGACGTAATCCACGATCT. PCR was carried out for the relative quantification of target gene copy numbers in relation to the *β*-actin transcript.

### 2.8. Blood Sampling

To examine the systemic response to wild-type BMSC transplantation, blood was collected from the inferior vena cava of anaesthetized rats at one day (1-day group; *n* = 6), four days (4-day group; *n* = 6), or one month (1-month group; *n* = 6) after the transplantation of wild-type BMSCs. The control group, which was injected only with PBS (control group; *n* = 6), was examined as well. The serum concentrations of blood urea nitrogen (BUN), creatinine (Cr), aspartate transaminase (AST), and alanine transaminase (ALT) were determined using automatic dry chemical analysis methods (VITROS 5600 Integrated System, Johnson, America). Routine blood test measurements were conducted with an automatic hematology analyzer (SYSMEX XN-1000, Japan).

### 2.9. Histopathology Evaluation

Tissue samples collected from the 1-day, 4-day, and 1-month groups were fixed in 10% formalin and subsequently embedded in paraffin following standard methods. Sections with a thickness of 5 *μ*m were cut and mounted on glass slides and then deparaffinized. Finally, the slides were stained with hematoxylin and eosin (H&E). The slides were relabeled using Arabic numbers, followed by double-blinded examination by two pathologists.

### 2.10. Statistical Analysis

All of the presented data are expressed as the mean ± standard deviation (SD). Statistical analyses were performed using SPSS version 22.0. Differences between different groups were examined using Student's *t*-test or analysis of variance. *P* values < 0.05 were considered significant.

## 3. Results

### 3.1. Transduction of BMSCs

**Figure 1:**
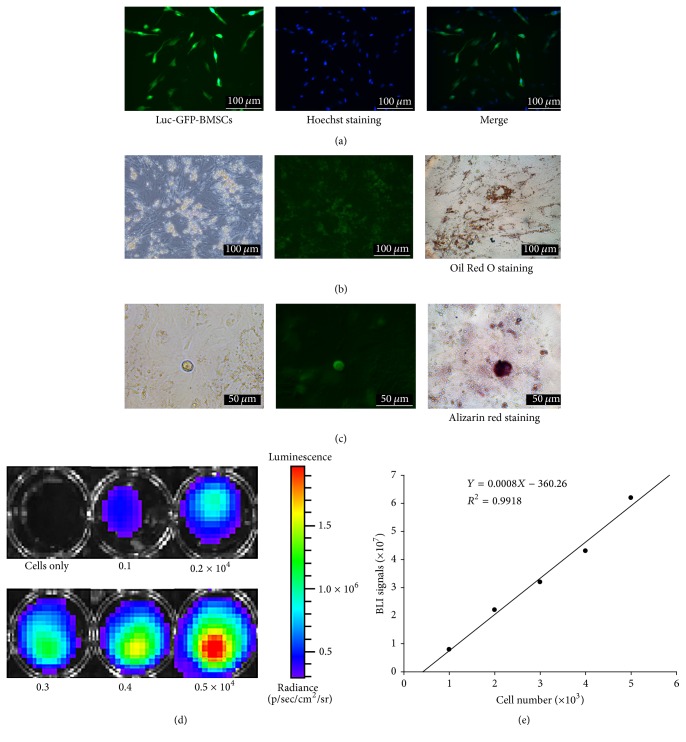
Characterization of rats treated with Luc-GFP-BMSCs. (a) GFP expression in Luc-GFP-BMSCs. The transduction efficiency was estimated by comparing the number of GFP-positive cells with the total cells via Hoechst staining. (b and c) The capability of Luc-GFP-BMSCs to differentiate according to an adipocyte or osteopenia pattern was verified by staining with Oil Red O and Alizarin red, respectively. After adipocyte and osteopenia differentiation, the cells still express GFP. (d) BLI of varying numbers of Luc-GFP-BMSCs* in vitro*. Representative images of at least three independent experiments. (e) Quantitative analysis revealed a strong linear relationship between cell numbers and the BLI signal (*R* ^2^ = 0.9918).

BMSCs were incubated in six-well plates at 1 × 10^5^ cells/well for 24 h, following infection with 4 × 10^5^ Lenti-Luc-GFP-lentivirus particles (MOI = 4). After 3 days of incubation, the transduction efficiency was approximately 76.45%, as evaluated under a fluorescence microscope (Figure 1(a)). There was no difference between the transduced BMSCs and wild-type BMSCs in terms of morphology. The expression of GFP was stable for at least 60 days under constant culture conditions.

### 3.2. *In Vitro* Characterization of the Pluripotential Capacity

To confirm the multilineage differentiation capacity of the Luc-GFP-BMSCs, the ability of Luc-GFP-BMSCs to differentiate into cells showing adipogenic and osteopenia patterns was investigated. Luc-GFP-BMSCs at passage 6 could differentiate into both adipocytes, as demonstrated by Oil Red O staining (Figure 1(b)), and osteoblasts, as assessed via Alizarin red staining (Figure 1(c)). Moreover, the differentiated cells still stably express GFP (Figures 1(b) and 1(c)).

### 3.3. *In Vitro* Imaging

The activity of luciferase was assessed through bioluminescence imaging (BLI) (Figure 1(d)). As shown in Figure 1(e), the imaging of different numbers of cells* in vitro* revealed a linear correlation between the BLI signal and cell numbers (*R* ^2^ = 0.9918), indicating that the reporter gene could be used for tracking and quantifying the transplanted BMSCs in small living animals.

### 3.4. Dynamic Distribution of BMSCs Monitored with an IVIS after Systemic Administration in Normal Rats

**Figure 2:**
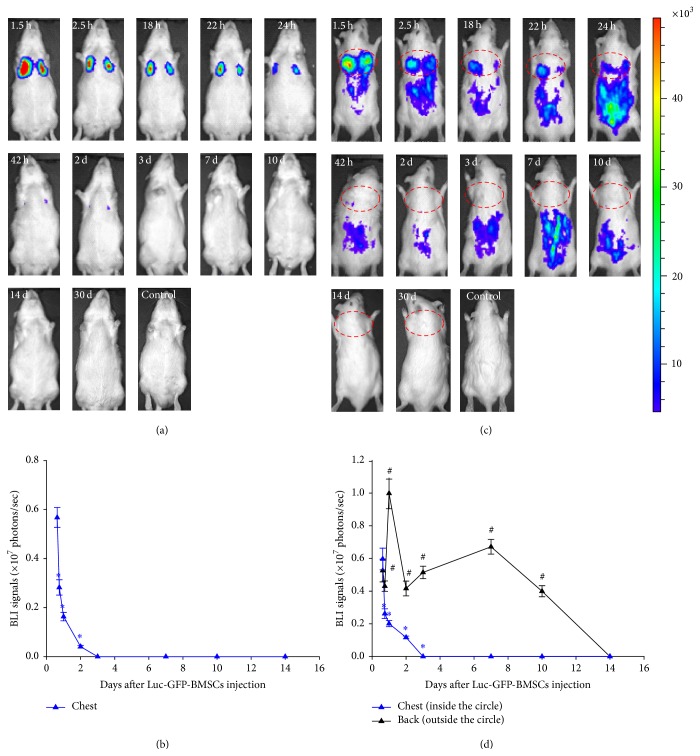
BLI of the transplanted Luc-GFP-BMSCs* in vivo*. (a and c) Luc-GFP-BMSCs suspended in PBS were injected into normal rats, while PBS alone was injected as a control. Cells were imaged at different time points, showing initial detention in the lungs and gradual concentration in the back, demonstrating that BMSCs may possess a tissue-specific binding capacity for some organs. However, the signals disappeared at day 14, revealing the final loss of transplanted BMSCs in normal rats. (b and d) Quantitative analysis of BLI signals in the chest and back. Bioluminescence activity steadily decreased over time in the chest. However, in the back, significant enhancement occurred at one and seven days after injection. ^*∗*^ *P* < 0.05 versus BLI signals in the chest at 1.5 h (*n* = 5); ^#^ *P* < 0.05 versus BLI signals in the back at 1.5 h (*n* = 5).

Next, we infused Luc-GFP-BMSCs into normal Wistar rats via tail vein injection and then dynamically monitored the bioluminescence imaging of luciferase activity with an IVIS in the following month. As shown in Figure 2, the BLI image results revealed that BMSCs delivered to fully immunocompetent allogeneic hosts predominantly resided in the lungs and lower back areas (Figures 2(a) and 2(c)). The BLI signals in the lung decreased over time and were absent at three days after injection* in vivo*. By imaging in both supine and prone positions, we found that the BMSCs migrated to the lower back after tail vein injection. There were two peaks of BLI signals observed in the lower back, at 24 h and seven days after vein injection. The imaging signals decreased over time and were completely absent 14 days after transplantation (Figures 2(b) and 2(d)). One month later, we still failed to find any signals in the animals.

### 3.5. Histological Distributions of the Transplanted BMSCs

**Figure 3:**
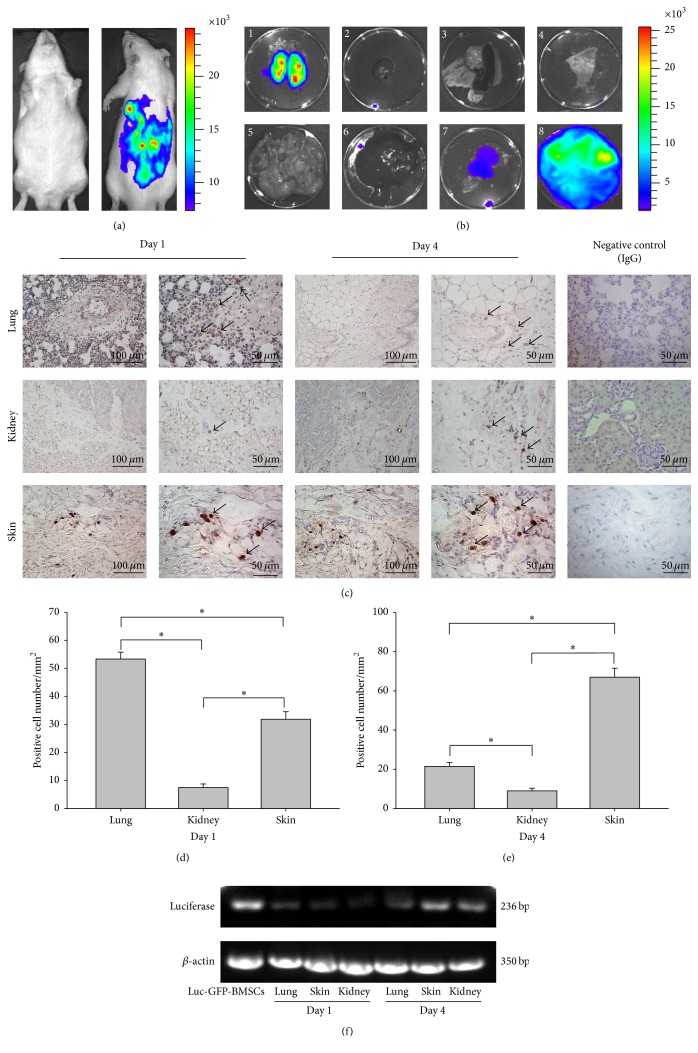
Distribution of the transplanted Luc-GFP-BMSCs within different organs. (a) BLI signals were diffusely distributed on the back* in vivo* at 4 days after injection. (b) IVIS photographs of dissected lungs (1), hearts (2), spleens (3), vertebral columns (4), intestines (5), livers (6), kidneys (7), and the skin of the lower back (8), showing direct confirmation of bioluminescence activity; Luc-GFP-BMSCs were localized to the kidneys, lungs, and the skin of the lower back (*n* = 5). (c) Immunohistochemically stained kidneys, lungs, and skin collected at 1 day as well as 4 days after injection. The specific position and positive cell counts were determined. (d and e) Positively stained cells in the lungs, kidneys, and skin were counted, and the migration of BMSCs appeared to be tissue associated over time. *n* = 5, and *∗* presents *P* < 0.01. (f) The expression of the firefly luciferase gene was detected via RT-PCR. *β*-actin was used as a loading control. Target gene expression was found in the lungs, kidneys, and skin of animals at 1 day as well as 4 days after injection. Representative images of at least three independent experiments.

To evaluate the distribution of Luc-GFP-BMSCs in different organs after injection, we injected the rats with D-luciferase intraperitoneally and isolated various rat organs, including the lungs, kidneys, heart, intestines, liver, skin of the lower back, spine, and spleen at four days after vein injection of Luc-GFP-BMSCs. The BLI signals were assessed in these organs, and the results showed that positive signals were detectable in the lungs, kidneys, and skin of the backs of normal rats (Figures 3(a) and 3(b)).

**Figure 4:**
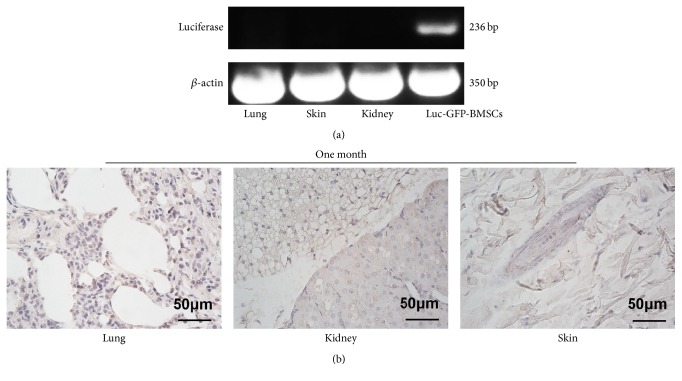
Detection of Luc-GFP-BMSCs in tissues at one month after injection. (a) The expression of the firefly luciferase gene was detected via RT-PCR. *β*-actin was used as a loading control. Target gene expression was not found in the lungs, kidneys, or skin. *n* = 5. Representative images of at least three independent experiments. (b) Immunohistochemistry detection of GFP. The absence of GFP-positive cells verified the BLI results at one month after injection (*n* = 5).

Furthermore, we detected GFP-positive cells in organs exhibiting BLI signals via immunohistochemistry to verify the advanced histological distribution of Luc-GFP-BMSCs. IHC was performed on the lung, kidney, and dorsal skin tissues. At one day after transplantation of Luc-GFP-BMSCs, GFP-positive cells were found in the conjunctive area between the blood vessels and alveoli. Although there were still BLI signals detected in isolated lungs at four days after injection, the IHC results showed that the positive cells were mainly located in the connective tissue and adipose tissue of the hilus pulmonis, but not the lungs (Figure 3(c)). Similarly, BMSCs were located in the connective tissue and adipose tissue of the renal hilum, but not the renal parenchyma. In the skin tissue, GFP-positive cells mainly appeared in the subepidermal connective tissue as well as the adipose tissue (Figure 3(c)). Consistent with the BLI results, the greatest number of GFP-positive cells was found in the lungs at one day after injection (Figure 3(d)), and four days later, the number of GFP-positive cells in the dorsal skin was greater than in other organs (Figure 3(e)). RT-PCR results also verified the presence of Luc-GFP-BMSCs in the above organ tissues: lung, kidney, and skin (Figure 3(f)). Furthermore, consistent with* in vivo* imaging result, BMSC colonization of specific organs was not found by RT-PCR and IHC detection (Figures 4(a) and 4(b)).

### 3.6. Safety Evaluation

**Figure 5:**
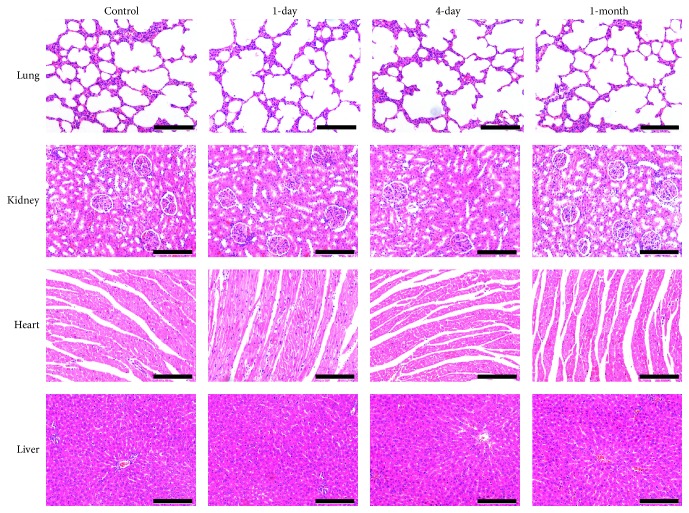
Light photomicrographs of tissue sections (HE). Compared with the control, there were no pathologic changes in the experimental groups. *n* = 6; scale bar = 100 *μ*m.

No death occurred in the experimental groups, and clinical signs of dyspnea did not appear throughout the experiment. The morphometric analyses failed to detect any tumors or neoplasms in all animals. HE staining demonstrated that there was no dysplasia in MSC homing organs in the 1-day, 4-day, and 1-month groups (Figure 5). Moreover, as shown in Table 1, there was no significant difference in the biological markers of the kidney and liver (*P* > 0.05) between the experimental groups and the control. Routine blood tests verified a reduction in the white cell count, especially for neutrophils (*P* < 0.05), resulting in a relative increase in the percentage of lymphocytes (*P* < 0.05) in the 4-day group compared with the control. However, one month later, the neutrophil count had returned to normal and was not significantly different than in the control (Table 2).

## 4. Discussion

Cell therapies are currently expected to provide cures for a wide variety of diseases as short effective routine therapies in clinical settings [17]. In clinical practice, in terms of the immune reaction, the perfect donor cells would be autologous [13]. However, autologous cells have obvious shortcomings. For example, in patients with diseases of the blood or immune system, their own cells are unsuitable for application. Due to showing low expression of immune antigens, allogeneic MSCs are an attractive cell resource for various complicated and refractory diseases [18]. The present study employed allogeneic cells, and we choose intravenous injection as the transplantation route to closely imitate clinical settings, as this route is commonly used in humans.

There is controversy about the homing feature of systemically administered BMSCs. The fate of BMSCs in living animals is related to many factors, such as their origins, the number of cells, and delivery routes. Tracking studies have demonstrated that transplanted human or allergenic BMSCs initially reside in the lungs and then egress to the liver and spleen in SCID mice [19, 20]. Eggenhofer et al. found that allergenic mouse BMSCs did not migrate beyond the lungs after intravenous infusion [21]. The differences in the distribution of BMSCs among these studies might be related to the origins of the cells, the host animal type, and the applied detection methods. Unlike the above studies, we injected allergenic BMSCs into normal rats (a larger animal subject). Our results showed that the majority of BMSCs localized to the lungs, kidneys, and loose connective tissue under the epithelia of the back after tail vein injection. In accordance with previous studies, a large number of IV-injected BMSCs were trapped within the first filtering organ rich in capillaries because the mean size of the suspended BMSCs is larger than that of capillaries [22]. In this work, we found that the BMSCs were inclined to undergo passive clustering after systemic infusion in the lungs and kidneys, but homing to the epithelia of the back may be an active phenomenon. BMSCs are not circulating cells, which must attach to the extracellular matrix for survival and growth. Accordingly, when such cells are disaffiliated from this context, they may be tracked using certain extracellular matrix-derived signals for survival. Regarding their secondary distribution, BMSCs mainly concentrate in loose connective tissue and adipose tissue under the epithelia. Even in the lungs and kidneys, they did not appear in the parenchyma, but in the connective tissue and adipose tissue of the hilus pulmonis and renal hilum at 4 days after injection.

By view from both supine and prone position, we firstly found that the dorsal skin is another organ attracting MSCs. IHC test showed that Luc-GFP-BMSCs locate in the loose connective tissue of the dorsal skin. Considering that BMSCs can easily differentiate into adipocytes* in vitro*, we hypothesize that the administered BMSCs are drawn by signals from the extracellular matrix of adipocytes and that the loose connective tissue is convenient for BMSCs to move through and proliferate. Different from the muscular tissue (heart and skeletal muscle), loose connective tissues have more space which may be suitable for the residing of MSCs. Moreover, dermal fibroblast secretes stromal cell-derived factor-1, a key chemokine which recruits circulating MSCs through the SDF-1*α*/CXCR4 pathway [23, 24]. As to the reason of BMSCs' selective recruitment to dorsal skin, we cannot give an exact explanation, which may need further research to answer this phenomenon in our following studies.

Previously, it was believed that MSCs repair damaged tissues through a transdifferentiation mechanism [25], and targeted delivery routes of the cells to injured organs would therefore be essential for effective therapy. These cells are also typically regarded as one of the notable landmarks in the progress of all forms of cell therapies. To cause greater numbers of MSCs to concentrate in injured tissues, researchers have attempted diverse injection strategies, leading to the detainment of MSCs in other target filtering organs. However, persistent engraftment and differentiation of implanted MSCs* in vivo* have rarely been detected. Many investigators have found that transplanted MSCs show short survival* in vivo*, and they exert a considerable influence on diseases that do not depend on the number of cells homing to the targeted organ [26, 27]. The life of transplanted MSCs* in vivo* is too short to explain the significant functional improvement of infarcted organs by differentiation. Thus, the routes for MSC administration do not appear to be as important in clinical applications. Thus, intravenous administration may be sufficient.

Our data from follow-up studies of transplanted BMSC survival using BLI demonstrated that BMSCs cannot survive for a long period. These results are similar to those of previous studies. There are three possible explanations for the observed localization of the administered BMSCs. (1) Mesenchymal cell adhesion to the extracellular matrix generates tensional integrity, which is a physiological cellular process that is necessary for cell differentiation, survival, and growth. In contrast, after administration and suspension in blood, MSCs lose their cell/matrix interactions, which will induce programmed death. This type of apoptosis is known as anoikis [28]. We believe that anoikis likely plays an important role in the disappearance of MSCs in this context because MSCs are not circulating cells and require extracellular matrix-derived signals for survival, and the deprivation of these signals in the vasculature might induce anoikis. (2) In the present study, we employed immunocompetent rats without the application of immunosuppressive agents during the whole experiment. The innate immune system, which is responsible for the removal of transplanted cells, may be activated by allogeneic MSCs. Some researchers believed that MSCs would not induce an immune response by themselves, but fetal bovine serum (FBS), which is widely used in cell culture and cannot be removed using phosphate-buffered saline, stimulates immunogenicity [29]. In a clinical setting, to avoid such risks, the adoption of autologous serum should be considered, as it has been found that autologous human serum results in more rapid expansion of MSCs [30]. (3) The kidney is a metabolic organ with abundant blood flow. It is known that the excretion of many drugs is closely related to renal function. Our data showed that BMSCs migrated to the kidneys and then disappeared from this organ. Another study [31] provided direct evidence that 1% of BMSCs that were present in the glomerular and peritubular capillaries were TUNEL positive. Together, these findings suggest that this process may contribute to the declining number of BMSCs after administration. Continued attrition of the very small number of cells eventually results in the disappearance of all cells. However, in this work, instead of the glomerular capillaries, we discovered BMSCs in the loose connective tissue around the peritubular capillaries. It is unknown if such a situation occurs occasionally or is related to different sampling times. Perhaps the kidney represents a secondary route of metabolism, but not the major one. Thus, larger, randomized, placebo-controlled preclinical animal trials need to be carried out to address the question of where MSCs are going.

The present study also investigated the short- and middle-term safety of allogeneic BMSC transplantation. Unlike pharmaceutical drugs, we are unable to produce MSCs in complete compliance with the relevant regulations in the laboratory [32]. However, we made all possible attempts to avoid potential adverse events after BMSC transplantation, such as bacteriological contamination, using a cell incubation period of less than 60 days and not exceeding 10 passages in culture. The experimental groups were followed up after one day, four days, and one month, and all animals exhibited an absence of tumorigenesis and injured tissues. Histological analysis of the experimental groups showed that all animals did not display any hyperplasia or tissue inflammation. There was no significant difference between the experimental groups and the control in terms of biological markers of the kidney and liver. Although infused MSCs were found to cause a reduction in neutrophils count at 4 days after injection, the sample sizes are too small to reach a definitive conclusion about whether allogeneic BMSC transplantation causes the white cell count to decrease. However, we think that care should be taken because of the observed phenomenon, and statistical analyses of large samples of blood cell count data associated with MSC-based therapies will be needed for future evaluations of long-term safety.

In summary, our results demonstrate that systemically administered BMSCs in healthy rats are short-lived and mainly migrate to the lungs, kidneys, and the skin of the lower back. Interestingly, in histological analysis, the BMSCs show lipophilicity. They collect in the adipose tissue, and a small number are located in the loose connective tissue around the blood vessels of the hilus pulmonis and renal hilum. The reasons for this distribution pattern are not fully understood. Safety assessment failed to detect any adverse effects in the animals over a short period. Thus, BMSCs may be an ideal candidate for cell-based therapy in preclinical animal studies and, subsequently, for clinical trials, but long-term follow-up safety assessments are needed.

## Supplementary Material

The marker expression of the BMSCs was analyzed by flow cytometry. The mesenchymal stromal cells expressed not hematopoietic markers but the well-known MSC markers.

## Figures and Tables

**Table 1:** Changes in the routine blood tests of the four study groups.

	Control	1-day	4-day	1-month
WBC (*∗*10^9^/L)	10.19 ± 3.42	6.95 ± 1.13	8.12 ± 1.81	7.49 ± 1.82
RBC (*∗*10^12^/L)	7.10 ± 0.85	6.57 ± 0.37	6.77 ± 0.20	7.43 ± 0.25
PLT (*∗*10^9^/L)	1020.50 ± 165.04	942 ± 133.95	1049.00 ± 83.606	936.17 ± 123.89
LYMPH (*∗*10^9^/L)	7.68 ± 2.40	5.55 ± 0.89	6.96 ± 1.51	5.80 ± 1.67
NEUT (*∗*10^9^/L)	2.02 ± 0.96	1.20 ± 0.28	0.97 ± 0.27^*∗*^	1.39 ± 0.25
LYMPH%	76.18 ± 5.20	79.98 ± 2.04	85.80 ± 2.50^*∗*^	76.87 ± 4.51

Control: animals injected with PBS only; 1-day: animals examined 1 day after transplantation of BMSCs (2 × 10^6^); 4-day: animals examined 4 days after transplantation of BMSCs (2 × 10^6^); 1-month: animals examined 1 month after transplantation of BMSCs (2 × 10^6^).

Results are shown as the mean ± SD (*n* = 6).

^*∗*^ *P* < 0.05 versus control.

**Table 2:** Serum AST, ALT, Cr, and BUN levels in the four study groups.

	Control	1-day	4-day	1-month
AST (U/L)	106.5 ± 22.28	124.60 ± 10.69	105.67 ± 18.46	99.17 ± 19.19
ALT (U/L)	34.00 ± 8.60	38.60 ± 5.18	34.50 ± 3.62	31.17 ± 4.12
Cr (*μ*mol/L)	30.67 ± 6.02	27.21 ± 3.56	29.67 ± 2.52	31.00 ± 2.37
BUN (mmol/L)	5.55 ± 0.49	4.76 ± 1.08	5.43 ± 0.70	5.38 ± 0.89

Control: animals injected with PBS only; 1-day: animals examined 1 day after transplantation of BMSCs (2 × 10^6^); 4-day: animals examined 4 days after transplantation of BMSCs (2 × 10^6^); 1-month: animals examined 1 month after transplantation of BMSCs (2 × 10^6^).

Results are shown as the mean ± SD (*n* = 6).
