# Supplementary material for: In Vivo Tracking of Systemically Administered Allogeneic Bone Marrow Mesenchymal Stem Cells in Normal Rats through Bioluminescence Imaging
Source: Stem Cells Int. 2016 Aug 17;2016:3970942. doi: 10.1155/2016/3970942 (PMC5005574; doi:10.1155/2016/3970942)
Supplement: Supplementary file 1 — The marker expression of the BMSCs was analyzed by flow cytometry. The mesenchymal stromal cells expressed not hematopoietic markers but the well-known MSC markers. [file 3970942.f1.docx]

**Supplemental Materials**

**
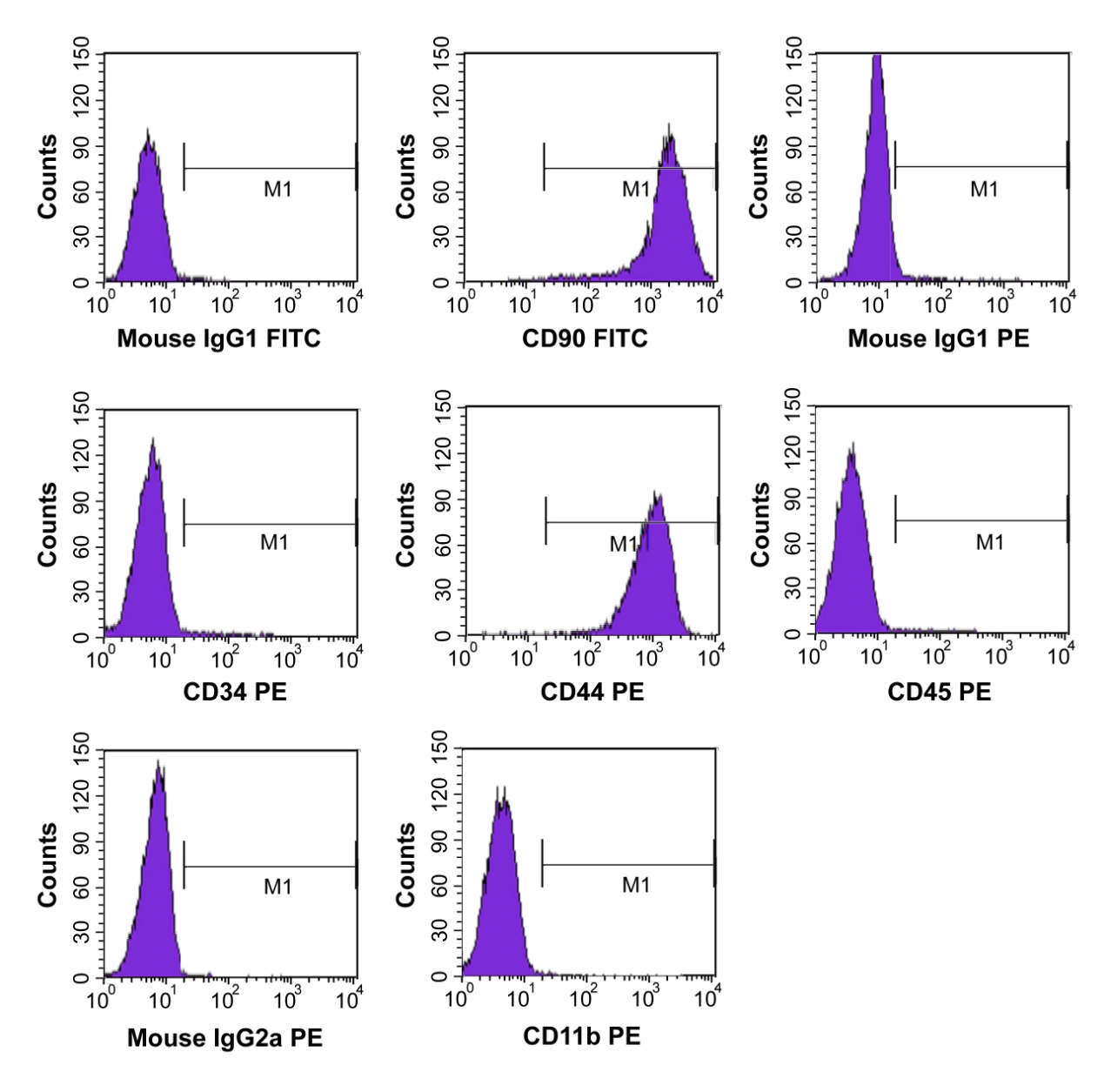
**

Figure S1. Flow cytometric analysis of Wistar rat BMSCs (provided by Cyagen Biosciences). Phenotypic markers were evaluated.
